# Deaths by Cholera in Boston

**Published:** 1849-11

**Authors:** 


*Deaths by Cholera in Boston.*—From the Boston Medical and Surgical Journal we extract the following statistics relating to the deaths by cholera during the .recent prevalence of the epidemic. The first death occurred on June 3d. During the month of June there were eight deaths. In July there were fifty-two deaths. In August, four hundred and eighteen. In September, one hundred and thirty-three. Total, six hundred and eleven.

In the cholera Hospital, it appears by the report of the physicians, that the whole number of admissions was two hundred and sixty-two, of whom one hundred and seventy-five died, or nearly sixty-seven per cent.

The birth places of the deceased, as near as could be ascertained, are as follows:—

Born in Boston, of whom many were of foreign parentage, 79; Massachusetts, out of Boston, 34; New Hampshire, 14; Maine, 16; Vermont, 5; Connecticut, 4; other American states, 11: Ireland, 379; Scotland, 12; England, 14; British American Provinces, 14; Germany, 3; France, 3; other foreign countries, 8; not ascertained, 15.—Total 611.

